# Professional obligations and the demandingness of acting against one′s conscience

**DOI:** 10.1136/jme-2024-110447

**Published:** 2024-12-24

**Authors:** Alberto Giubilini

**Affiliations:** Uehiro Oxford Institute, University of Oxford, Oxford, UK

## Abstract

Conscience is typically invoked in healthcare to defend a right to conscientious objection, that is, the refusal by healthcare professionals to perform certain activities in the name of personal moral or religious views. On this approach, freedom of conscience should be respected when the individual is operating in a professional capacity. Others would argue, however, that a conscientious professional is one who can set aside one′s own moral or religious views when they conflict with professional obligations. The debate on conscientious objection has by and large crystallised around these two positions, with compromise positions aiming at striking a balance between the two, for instance, by arguing for referral requirements by objecting healthcare professionals.

In this article, I suggest that the debate on conscientious objection in healthcare could benefit from being reframed as a problem around demandingness rather than one about freedom of conscience and moral integrity. Being a professional, and a healthcare professional specifically, typically requires taking on additional burdens compared with non-professionals. For instance, healthcare professionals are expected to take on themselves higher risks than the rest of the population. However, it is also widely agreed that there are limits to the additional risks and burdens that healthcare professionals should be expected to take on themselves. Thus, a question worth exploring is whether, among the extra burdens that healthcare professionals should be expected to take on themselves as a matter of professional obligation, there is the burden of acting against one′s own conscience.

## Introduction

There has been a long-standing debate around the limits of a duty of care by healthcare professionals, that is, about how far healthcare professionals’ duty of care can go before risks and burdens are considered too great and beyond the call of duty, for instance, during epidemics or other public health emergencies. There has been a long-standing debate about conscientious objection in healthcare, that is, on whether and when healthcare professionals should have the right to object to delivering some types of healthcare on the basis of their moral opposition to them, for instance, in the case of conscientious objection to abortion. For some reason, these two debates have largely run in parallel, with some,^1^ but not much explicit overlapping. Granted, the idea that acting against one’s conscience could be so emotionally or even physically burdensome to be professionally overdemanding implicitly underlies a lot of the positions in favour of a right to some form of conscientious objection in healthcare. However, there is a question as to whether, or to what extent, it could be used as an argument in support of a right to conscientious objection in healthcare.

Most people tend to agree that healthcare professionals’ duty of care requires them to take on themselves additional burdens, compared with non-professionals, such as doctors attending to emergencies even when off duty. I suggest that demandingness can represent the common conceptual currency that both parties would likely consider ethically relevant for establishing professional obligations. Thus, I suggest that the debate on conscientious objection in healthcare could benefit from being reframed as a problem around demandingness rather than one about freedom of conscience and moral integrity. Within this framework, the relevant question becomes the following. If healthcare professionals are expected to take on themselves additional burdens, compared with non-professionals, in caring for patients, do these include the burdens of acting against their own conscience?

In this article, I will use the HIV epidemic at the end of the last century as a case study to illustrate the debate around demandingness of professional obligations—though my analysis will only be focused on very few aspects of the issues that that epidemic raised for professionals and professional organisations. There are a couple of reasons for this choice. One is that, especially in its initial phase, the debate around the response to the HIV epidemic brought to light the need to set criteria for the limits of professional duties of care. The second is that, inadvertently, it also highlighted the potential tension between professional organisations’ stance on duty of care and on conscientious objection.

However, I will not say much about what duties professionals had during that or other epidemics. Here, I am mostly interested in the conceptual connections between the issues of demandingness and of conscientious objection than in providing an answer to the question of what healthcare professionals should do on any specific occasion.

Two clarifications are in order, before I proceed with my analysis. Some would claim that the same considerations about conscientious refusal to providing certain services extend to refusal to providing referrals to some colleagues, since the two involve comparable levels of complicity (for instance in the Catholic doctrine of formal cooperation in wrongdoing), while some would contest this equivalence.^2^ Here, I want to remain neutral on this point, and the reader can understand conscientious objection broadly to include objections to referrals or narrowly to only include objections to service provision. This should not make a difference to the conceptual points I will be making (though it might make a difference to the practical implications, eg, in terms of assessing levels of demandingness of the two options). Similarly, I will not address the point that there might be a societal interest in allowing conscientious objection—for instance, to ensure that enough people will want to enter the profession despite objecting to specific procedures, so as to guarantee enough supply of workforce in the healthcare sector. There might well be reasons for accommodating conscience that do not depend on principles of freedom or of duty of care, but these are beyond the scope of this paper.

### Framing The Problem: Conscientious Objection, Moral Injury, Moral Distress

Although I have just suggested that framing the debate on conscientious objection in healthcare in terms of the demandingness of acting against one’s own conscience could be helpful, it might also be seen as missing the point of what is at stake in this debate. This approach would frame the problem in the merely quantitative terms of the amount of burdens that doctors should be expected to bear, rather than in the qualitative terms of the value of conscience, freedom of conscience or moral integrity. This might not do justice to what is often taken to be at stake in the debate over a right to conscientious objection. Someone might say that a principle of freedom of conscience in the healthcare profession is worth defending because of the value of conscience itself,^3 4^ or the value of moral integrity itself,^5 6^ or the importance of doctors’ conscientious judgements in pursuing the proper goals of healthcare,^7^ regardless of how burdensome it is for healthcare professionals to be forced to do something contrary to their conscience.

However, this framing is not innocent. To claim that the debate should be framed in terms of the proper place of freedom of conscience and the value of moral integrity already presupposes a stance that those who argue against conscientious objection would not accept. That is, it presupposes that freedom of conscience is compatible with professional obligations, and the only question is how to reconcile them. To someone who is opposed to a right to conscientious objection, appeals to freedom of conscience or moral integrity are questions begging. One of the problems with conscientious objection—so they would argue—is precisely that the value that freedom of conscience and moral integrity have in society at large, and the arguments for protecting them through legal and political means, cannot be simply transferred into professional settings—at least in the case of freely chosen professions.^8 9^ That is because professions have their own internal norms that are different from societal norms. For instance, a doctor has a duty to alleviate a patient’s suffering that is different from the duty I have to alleviate someone else’s suffering. One has the freedom to decide whether or not to enter a profession, but not to act on personal idiosyncratic values once in it, at least according to those who oppose a right to conscientious objection.^10^

There is a richer spectrum that includes different middle-ground positions—such as those whereby conscientious objection should be tolerated in the name of freedom of conscience as long as the objection meets some standard of reasonability^11^ or an effective referral is in place.^12 13^ I am not denying that any of these positions could be right. But the issue framed in this way is unlikely to be settled not just within academic debates (where hardly anything ever gets settled), but also in societal and policy discussions about the duties and the rights of healthcare professionals.

It seems the framing itself precludes examining the issue from a perspective that could, if not settle the matter, at least bring into the picture a wider range of considerations. Perhaps when we reach an impasse, it is helpful to look at the problem from a different perspective.

It is now acknowledged that so-called ‘moral distress’ and ‘moral injury’ are significant risk factors for burnout.^14^ ‘Moral distress’ is a phenomenon that has been identified as an important factor contributing to psychological distress in nursing staff.^15–17^ It usually arises ‘from the discrepancy between knowing the ethically appropriate course of action yet being unable to pursue that course of action because one’s behaviour is limited by institutional constraints’.^18^ Moral injury—a concept that started to be used in the military context to describe the experience of some soldiers who witnessed the atrocities of war—is traditionally difficult to define and consensus on a definition is lacking.^19 20^ While a comprehensive definition is beyond the scope of this article, it is useful to provide a scientific and a non-scientific understanding of the term as indicative of the ways in which infringements of conscience can be framed as moral injuries. A scientific definition of the concept was first provided by Litz *et al*,^21^ who conceptualised moral injury in terms of a symptomatology including posttraumatic stress disorder, acts of self-harm, feelings of hopelessness, following ‘perpetrating, failing to prevent, bearing witness to or learning about acts that transgress deeply held moral beliefs and expectations’.^21^ In a more philosophical fashion, moral injury can be understood as a ‘character wound’ caused by one’s perception of having betrayed justice.^19 20^

One author, writing about moral injury in the military context, has recently defined it as the ‘aggravation of the moral emotions—guilt, remorse and shame—to the point of personal dysfunction’.^22^ It is not difficult to see how moral injury as briefly presented here is a likely consequence of acting against one’s conscience, including in the healthcare context—at least if by conscience we mean a set of strongly held moral beliefs, or a commitment to such beliefs, that is constitutive of someone’s identity.^23^ The literature has identified the source of moral injury for healthcare professionals mostly in the experience of suffering and the feeling of not being able of doing enough to alleviate it, including providing care to patients knowing they won’t benefit from it.^15^ However, what has been called ‘potentially morally injurious events’ have also been characterised more broadly as ‘perpetrating, failing to prevent, or bearing witness to acts that transgress deeply held moral beliefs and expectations’.^21^ Transgression of one’s conscience by performing oneself those acts one deeply opposes morally, such as providing an abortion, cannot but exacerbate the phenomenon.

If one wants to claim exemptions on the basis of conscience, this seems to be a more promising path to pursue when engaging in discussion with opponents. When it comes to moral injury, it seems the issue is no longer about fear or risks for one’s physical health, but it is still about health and well-being nonetheless, in the same way as exposure to risky pathogens or other occupational hazards are. In fact, as one reviewer of this article notices, unlike other types of exposure that merely involve risks of harms—such as exposure to an infectious pathogen—moral injury is at least for some the result of exposure that is itself harmful. In this sense, it is more similar to radiation in a surgical theatre, which one cannot avoid—even with protection—when entering the theatre in the way you can avoid pathogens (I am grateful to an anonymmous reviewer for this analogy).

Moral injury, as we have seen, has started being discussed as a pathological category. It is reasonable for a healthcare professional to take some, but not too much. This would not address all the issues that conscientious objection traditionally raises, but it might go further than appeals to freedom of conscience or moral integrity in providing a shared basis for discussion

### The Call of Duty: The Case of the Hiv Pandemic and the Social Contract

Every time healthcare professionals treat someone with a communicable disease, they are exposed to some risk. That risk can be higher than the risk each of us is expected to take on ourselves in caring for strangers. Yet, many of us would think that healthcare professionals do have an obligation to treat and therefore to take risks that the non-professional person normally does not have. That is just part of what being a healthcare professional entails. This difference between professional and non-professional obligations makes it difficult to establish what an acceptable level of burden or risk is for healthcare professionals. Quite simply, we cannot use our intuitive standard of moral obligations as a benchmark. The same is true, of course, for other professional categories, such as firefighters, police officers, or lifeguards.

The HIV pandemic in the ‘80s the ‘90s of the last century provides a good example of how, in times of crises, the question of the call of duty requires some precise answer. The question at that time was asked not only regarding the demandingness of taking on (alleged) additional risks of infection by healthcare providers, but also regarding the demandingness of the emotional burden involved, which seems closer to the type of burden involved by moral injury and moral distress described above. In 1986, the American Medical Association (AMA) issued a statement declaring that physicians would not be required to treat HIV-positive patients if they were not ‘emotionally able to do so’.^24^ However, this statement was soon overridden by a new one, according to which ‘a physician may not ethically refuse to treat a patient whose condition is within the physician’s current realm of competence solely because the patient is seropositive’.^25^ Indeed, the requirement that healthcare professionals take on themselves additional risks in emergency situations regularly appears in professionals’ codes of practice and position papers.

Many see such obligations as stemming from a social contract grounding the scope of the healthcare profession.^26 27^ In this view, healthcare professionals have an obligation to take on significant additional risks in virtue of a contract with society, which healthcare professionals implicitly accept when they choose to enter the profession.^27^ Of course, the same contract binds society—through appropriate investments—to minimise as much as reasonably possible the risks for healthcare professionals, for instance, by providing adequate personal protective equipment. Unfortunately, this will often not eliminate the additional risks. When it comes to the healthcare profession, the social contract is the same one whereby society grants healthcare professionals monopoly rights over the provision of certain services, in return for the provision of those services that society deems appropriate.^26^

In the strict sense of the term, a professional is someone who belongs to a category of workers with societal licence—and, therefore, a societal monopoly—to operate in a certain field, on the basis of the social recognition of certain skills (certified, for instance, through academic credentials).^28^ According to Rosamond Rhodes,^26^ “[b]ecause society has granted medicine a legal monopoly over the use of its knowledge, powers, privileges, and immunities, the profession and its members are required to provide treatment for everyone who needs it and who wants to avail themselves of the services.”

While the implication that Rhodes derives is questionable, the premise expresses well the idea behind the social contract approach to healthcare professionals’ special obligations and the related idea that healthcare professionals are patients’ fiduciaries.^29^ On the same social contract view, those special obligations are also grounded in the fact that ‘the profession has flourished due to socially negotiated promises to be available in such times of duress’.^27^ In this view, given the contractual nature of the monopolistic rights of healthcare professionals, taking on oneself higher risks and burdens could be seen as a way of ‘strengthening the professions standing in a contractual public trust’.^27^

What kind of burdens and risks do these special obligations actually include? It is interesting to note that according Fourth Edition of the American College of Physicians Ethics Manual in 1998, which explicitly referred to the risks of ‘potential occupational exposure such as HIV’, ‘the denial of appropriate care to a class of patients for any reason, including disease state, is unethical'. However, this statement, is followed, just a couple of pages after, by this point:

The ethical duty to disclose relevant information about human reproduction to the patient may conflict with the physician's personal moral standards on abortion, sterilization, or contraception. A physician who objects to these services need not become involved, whether by offering advice to the patient or by involvement in a procedure”.^30^

So it seems it is actually not the case that denial of appropriate care to a class of patient for any reason is unethical. Reasons of conscience, apparently, are not included. Is there a contradiction in this document? Why is conscientious objection treated differently? If it is wrong to deny appropriate care to a class of patients for any reason, it is wrong to deny appropriate care to a class of patients for reasons of conscience. Here, I am interested in how that differential treatment could be justified from an ethical point of view, rather than in what the intention or justification behind that principle actually was from a historical point of view. That is because I am interested in whether there can be a common currency for the ethical justification of professional obligations, or lack thereof, in the case of risks from exposure to pathogens and in the case of a conscientious objection.

One might ask whether the differential treatment can be justified by appealing to different implications of the same principle—that is, that physicians cannot be expected to provide services that are too demanding, but acting against one’s conscience is more demanding than occupational exposure to HIV (assuming these are commensurable); or whether there are two different principles at stake that are relevant in the two cases—that is, that physicians cannot be expected to provide services that are too demanding in the case of treating HIV-positive patients, and a principle of freedom of conscience in the case of conscientious objection. If the latter were true, then acting against one’s conscience would violate the relevant principle for that type of case, but occupational exposure to HIV would not violate the relevant principle for that other type of case.

However, the latter option seems to offer an arbitrary justification (and so in a sense no justification at all). Why wouldn’t a patient’s HIV status concern personal moral beliefs, and therefore conscience, as well? And therefore, why would a principle of freedom of conscience not protect a right to refuse to provide care to HIV patients, if it does protect a right to conscientious objection? After all, one might well think that HIV status is morally problematic when it is the result of certain types of conduct that one morally condemns, such as gay sex. If we thought the value of freedom depended on what one does with it, or the views one expresses, then we would be appealing to some other criterion than freedom of conscience. The value of freedom would be, at best, instrumental – freedom would be valuable when used to pursue good ends and questionable when used to pursue questionable ends. The moral evaluation would be shifted from freedom to those ends. It does not seem the strategy of using freedom of conscience itself to explain why healthcare professionals have a duty to care HIV-positive patients but not a duty to provide abortion is available to those who want to avoid the tension in that document and defend a right to conscientious objections.

Thus, if the two options above were the only available options, one would be left with the first one. The differential treatment in the American College of Physicians Ethics Manual concerning duty of care would be explained precisely by the hypothesis I am formulating in this article. To restate it, physicians cannot be expected to provide services that are too demanding, but acting against one’s conscience is more demanding than occupational exposure to HIV (assuming these are commensurable), and indeed, beyond the acceptable demandingness threshold (wherever). That is, a defence of conscientious objection could be based on an argument about the demandingness of acting against one’s conscience, rather than an argument about freedom of conscience.

However, other strategies might be available to those who want to defend a right to conscientious objection and indeed might justify the differential treatment in that Manual. One appeals to discrimination, the other on what counts as appropriate healthcare. Let me consider those options in order.

First, as mentioned, that principle refers to ‘classes of patients’ because it is meant to avoid discrimination in the delivery of healthcare. The reference there was to discrimination against HIV-positive patients, but the point is relevant to discussion of conscientious objection because discrimination against women is a reason sometimes used by those arguing against a right to conscientious objection abortion by healthcare professionals. Because HIV was more prevalent among gay men, there was a concern that refusal of care would be or express some form of discrimination on the basis sexual orientation. I want to suggest that, with regard the risk of discrimination, refusal of treating HIV-positive patients and conscientious objection are on equal footing. If one is discriminatory, so is the other. If one isn’t, neither is the other.

It is debatable whether conscientious objection amounts to a form of unfair discrimination. A behaviour that results in the differential treatment or differential outcomes for a class of people (say, women) is not necessarily a form of unfair discrimination. Conscientious objection is raised to a broader range of services than abortion or reproductive healthcare. Even if it results in differential treatment in one type of cases, we cannot make ethical arguments around conscientious objection by generalising from that type of case only. Objecting to providing euthanasia, for instance, would not result in sex-based differential treatment. Many emphasise the fact that conscientious objections are typically put forward against procedures, not against patients or classes thereof.^31^ The justification for it appeals to principles such as freedom of conscience, tolerance, pluralism, professionals’ autonomy in judging clinical cases, and so on. Neither the target nor the justification of the objection is about a class of patient. That women happen to be the only group affected by conscientious objection to abortion is incidental. Quite simply, there is no way conscientious objection to abortion could affect men. If, when putting forward a conscientious objection to abortion, one does not have the choice of targeting a group rather than the other, on some views the fact that only one group is targeted is not a form of unfair discrimination. On this account of discrimination, the same could be said about the treatment of HIV-positive patients. One could argue that the refusal is based on a refusal to take on the risk of infection, and it just so happens that HIV is more prevalent among gay men (if and when it actually is). The two cases are on equal footing if we assume an account of discrimination based on the intention behind the refusal to provide healthcare. We might reject this account and argue that what matters is the outcome—more gay men and more women would be affected by the refusal to provide healthcare to HIV-positive patients and abortion, respectively. If so, both would be cases of discrimination. For the purpose of this paper, the relevant point is that, on either interpretation, refusal to provide care to HIV-positive patients and conscientious objection are on equal footing, and therefore, appeals to discrimination cannot justify their differential treatment.

Alternatively, one could go down the ‘appropriate healthcare’ route. Denying care is deemed unprofessional when care is ‘appropriate’. Perhaps, then, one could justify the differential treatment by arguing that treating HIV-positive patients is appropriate healthcare, but providing abortion, generally speaking, is not. This is relevant to discussion of conscientious objection because often the procedures to which healthcare professionals more commonly object—most notably abortion—are not considered by defenders of a right to conscientious objection to be appropriate healthcare.^7^ Does this type of consideration justify treating refusal of care on the basis of health risks and refusals on the basis of conscience differently? I will suggest in the next section the answer is negative.

### Conscience, Risks, and Appropriate Healthcare

What counts as appropriate healthcare is up for debate. For example, one seemingly plausible view is that appropriate healthcare is the one that benefits patients while minimising harms, or the one in the patient’s best interest. Call this an interest-based account of the scope of healthcare. Determining what counts as appropriate healthcare is an ethical decision about what matters in healthcare delivery, and therefore, what healthcare professionals ought to pursue. But this criterion would be too broad. Not everything that is in the patient’s best interest and that a healthcare professional is qualified to provide ought to be provided as a matter of professional obligation and is appropriate. Most obviously, there are ethical constraints posed by fair allocation of scarce healthcare resources. The problem is that if we start adding ethical considerations to what counts as ‘appropriate’, we are narrowing down the concept in ways that inevitably lead to more disagreement—including the disagreement around the role of conscience here under examination. Such qualifications might include the morality of specific practices, such as abortion, and the ethics of requesting healthcare professionals to do something that is very demanding for them, either in terms of risks or in terms of infringements of their conscience. Whether caring for HIV-positive patients is appropriate healthcare would depend on ethical issues around the legitimate level of risks healthcare professionals should be expected to take on themselves. When HIV first appeared, the answer to this question was less obvious and more open to disagreement than it is now.

While most people would agree that only appropriate healthcare should be provided by healthcare professionals, many would disagree about what counts as appropriate according to these ethical criteria. Quite simply, people disagree about ethics. If you think abortion is immoral, you might still think that doctors should deliver appropriate care, but also that abortion is not appropriate care. Thus, to say that all and only appropriate care ought to be provided as a matter of professional obligation begs the question of what ethical considerations should be used to determine what is ‘appropriate’. Perhaps we should acknowledge that the issue of what counts as appropriate healthcare is bound to be subject to irresolvable disagreement and that, for the purpose of setting the boundaries of professional obligations, we should rely on other criteria—such as what the law or professional codes of practice permit, without assuming that that coincides with ‘appropriate’ healthcare. Again, what matters for the purpose of our discussion is that the limits of a duty of care in terms of taking on oneself risks of infectious diseases and acting against one’s conscience are on equal footing when it comes to establishing appropriate healthcare: they both beg the question of what criteria should be used and in both cases they require a discussion that does not simply assume what counts as healthcare. Thus, appealing to what counts as appropriate healthcare cannot justify the differential treatment.

Now, a defender of conscientious objection could reply that the autonomy of a healthcare professional, as guaranteed by the protection of healthcare professionals’ freedom of conscience, is indeed necessary to determine whether any intervention is appropriate healthcare. Cavanaugh, for instance, in making his case for freedom of conscience in healthcare delivery, wrote that

That aspect of one’s conscience that one’s profession forms and upon which one relies as a practitioner amounts to the professional facet of one’s conscience. […] in keeping with the internal ethic, one’s professional conscience (if I may so speak) makes unique demands on one’s conduct. Demands distinct from the morals one shares with others not of one’s profession.^32^

Notice, however, the shift from ‘conscience’ to ‘professional conscience’. An opponent of conscientious objection would not deny that healthcare professionals should operate according to their own professional conscience. But that is a conscience that is informed, as Cavanaugh himself acknowledges, by the ‘internal ethic’ of the profession. That might well be different from one’s own morality as dictated by one’s own conscience. Which brings us back to the issue that I raised above: who decides, and according to what standards, what the internal ethic of the profession should be?

This is a difficult question. Let me try with a different one: who should not decide what the ethics of the profession should be? What counts as appropriate healthcare cannot unilaterally be decided by any individual healthcare professional when faced with a patient on the basis of their own personal moral views. A shared standard for the profession is required. What that standard should be is a decision of the profession as a collective, for example, through codes of practice by relevant professional bodies, in negotiation with the society which bestows to professional monopoly rights, for example, through legislations approved by elected representatives that permit or restrict certain procedures. This would allow some consistency, at least at the local level, about what being a professional entails and what can be expected of professionals. Such consistency would, in turn, prevent healthcare professionals from unilaterally deciding the best course of action not on the basis of clinical criteria and of consideration for a patient’s best interest, but on one’s own moral views or preferences. There can be disagreement about who should be involved in this discussion and who gets to decide, but why should it be assumed that it should be up to an individual professional’s conscience? It is true that many would think that judgements of conscience are not separable from judgements of best interest^7^—after all, my conscientious judgement against, say, euthanasia is also a judgement that euthanasia is not in a patient’s best interest. However, the current paradigm of medical and professional ethics is one centred on patient autonomy, whereby the patient is the best judge of what is in his or her best interest, provided the decision counts as autonomous.

Constraints on autonomy do and should exist. A patient’s view of what is best for themselves or, for that matter, of what counts as appropriate healthcare is as constrained by professional and other ethical requirements as is the conscience of healthcare professionals. But the point is precisely that in either case such constraints are not related to the professional’s own judgement of conscience about best interest, which would reintroduce a form of paternalism not consistent with current codes of practice and principles of medical ethics.

Interestingly for the type of analysis and analogy presented here, Rhodes has made an analogous point regarding professional obligations to take on oneself additional risks. As she put it,

[t]here are certainly limits to professional responsibility, and risk exposure is a relevant consideration. But, as with other matters of professional ethics, the limit on what counts as an acceptable risk is a matter that must be settled by a determination from the profession rather than each individual physician making a personal judgment. Courageous doctors do not have greater professional responsibilities than timid ones. Every similarly exposed medical professional should have the same duty in a comparable situation (p.80)^26^

Analogously, one might suggest, healthcare professionals with more resilience or whose moral views do not oppose abortion cannot be expected to have greater professional responsibilities than, say, religious doctors. Responsibilities need to be established on independent grounds.

In the case of HIV, the professional consensus is that providing medical care to infected patients is a professional responsibility of each individual healthcare professional. This view is justified not because denying such care would be discriminatory—we have seen that that is not a good reason. It is justified because that counts as appropriate healthcare by the standards of the profession and because risks and burdens are not considered by the profession or by the employer significant enough to exempt from professional duties of care. Analogously, the ethics of abortion provision by healthcare professionals—or indeed the provision of any other medical service—should be a judgement of the profession or of the society that licences the profession.

This is not the same as to say that whether and when abortion is morally acceptable is a matter of professional judgements or legal decisions, of course. The morality of abortion involves dimensions that are broader than healthcare ethics. The fact is, healthcare professionals, qua professionals, have no special authority to establish whether the moral wrongness of abortion is such that its provision would count as inappropriate healthcare. As individuals, they might be subject to the authority of their own conscience, among other things. That is a private matter. However, the exercise of one’s profession is not a private a matter. If it is a matter of conscience, it is a matter of professional conscience, which is conscience informed by professional standards, including professional ethics codes and legislation determining who is eligible for what medical services. In the strict, technical sense in which they are professionals—which involves monopoly rights and societal licence and public oaths—the exercise of their profession involves responsibilities towards patients, professional bodies, and the society that warrants someone the status of a professional.

### The Burden of Acting Against One’s Conscience

Now, the alternative that remains on the table is that the limit of professional obligations, both in the case of treating infected patients and providing services one morally objects to, is determined by the demandingness of the activity. There is something to say for this option, although way more than the space remaining for this article would allow.

It would certainly be inconsistent for someone to accept that abortion is part of one’s professional obligations just because someone else with the relevant authority (the profession, society, the legislator, some ethicist) has made a decision to that effect, and at the same time morally oppose it as a matter of conscience. Indeed, the reason someone conscientiously objects to abortion is precisely that this person believes abortion is not appropriate healthcare, given its moral dimension, and therefore, should not be part of one’s professional obligations. But it would not be merely inconsistent. It would be extremely burdensome emotionally. Appeals to principles of freedom of conscience and to moral integrity overshadow this aspect. They shift the focus away from something that is typically not up for debate, that is, that there is a threshold of risks and burdensomeness after which healthcare professionals no longer have a professional duty of care.

The issue of additional risks and burdens that healthcare professionals are expected to take on themselves is often discussed, both in the academic literature and in professional codes of ethics, with reference to emergency situations. Opinion 9.067 of the AMA Code of Medical Ethics, for instance, states that “[b]ecause of their commitment to care for the sick and injured, individual physicians have an obligation to provide urgent medical care during disasters. This ethical obligation holds even in the face of greater than usual risks to their own safety, health or life”.^33^

However, when it comes to non-emergency circumstances, codes of ethics tend to grant healthcare professionals more freedom to refuse treating patients. For instance, Principle VI of the same AMA Code of Medical Ethics states that “A physician shall, in the provision of appropriate care, except in emergencies, be free to choose whom to serve … and the environment in which to provide medical care.”^33^ This seems to contradict a duty of care of medical professional codes. But it is important to place this principle in its historical context. The principle was introduced in 1980, when it was drawing on a previous formulation, in turn referring back to the formulation of the 1958 Code, which stated that ‘A physician is free to choose whom he will serve. He should, however, respond to any request for his assistance in an emergency or whenever temperate public opinion expects the service’ (as reported by Clark,^27^ emphasis added). This seemed to give more weight to the societal expectations towards healthcare professionals, regardless of the existence of emergencies. Indeed, as Clark notices, ‘In the 1912 Code, the duty to respond to ‘temperate public opinion’ is earmarked in the index with the imperative phrase, ‘Public opinion to command service’.^27^ The transformation seems to suggest that the AMA moved away from the duty to prioritise societal and patient interests which is grounded in the monopoly argument to become more protective of physicians’ interests and autonomy.

I quoted above Rhodes writing that ‘the profession and its members are required to provide treatment for everyone who needs it and who wants to avail themselves of the service’. This cannot be true. Giving more weight to physicians’ own interests as the AMA Code of Ethics did serves as a warning against obviously implausible implications of requirements such as this one. If there is an outbreak of smallpox, which comes with one in three chances of death if infected and risks of permanent damages including blindness for survivors, arguably the duty of care is not the same as a doctor would have if there was an outbreak of the seasonal influenza. A fundamental principle in the negotiation of professional obligations is to protect the safety of professionals as much as reasonably possible and taking into consideration interests and legitimate claims of patients. A difficult balance to strike indeed. The same consideration uncontroversially applies to other professions entailing risks. A lifeguard might be required to take on additional risks and burdens to save a drowning person, but not the burden of, say, saving a swimmer from the mouth of a white shark. Indeed, on some view, requiring healthcare workers to take on themselves the additional risks entailed by a pandemic of a severe disease like SARS or influenza is like requiring a lifeguard to save a swimmer from the mouth of a great white shark.^34^

In a social contract framework, the interests of healthcare professionals set reasonable limits to such expectations. There is a debate to be had about where to draw that line. But as the discussion on the HIV epidemic above exemplifies, and indeed as the broader academic and professional discussion around a duty of care shows, we are already having that discussion in a certain form, and we are having it on the basis of more widely shared assumptions than in the case of the debate on freedom of conscience. Indeed, that is a long-standing, constantly evolving debate, whose central premise is widely agreed on there is a limit to the burdens healthcare professionals should be expected to take on themselves. There is no good reason why this debate should not incorporate, within this widely shared assumption, a discussion about whether the mental health cost of acting against one’s conscience, framed in terms of moral injury and moral distress, should be factored in when setting the boundaries of a duty of care. This discussion would not need to appeal to principles or values such as freedom of conscience and moral integrity, which are contested within healthcare. If there is a mental health cost in providing healthcare in certain circumstances, and if that is significant enough, then there is a debate to be had about whether mental health burdens and risks should be included among the risks and burdens that are already agreed to set limits to healthcare professionals’ duty of care. And as part of this debate, the question of whether the mental health costs of acting against one’s conscience should feature, and should be treated in the same way as other types of mental health costs and indeed as other types of health risks and burdens.

There is disagreement on where exactly to draw a line, but not on the fact that there is a line to be drawn, after which risks and burdens for healthcare professionals are beyond the call of (professional and ethical) duty. The focus on moral distress and moral injury as criteria to limit a duty of care does not address the point that entering the healthcare profession is a free choice, and therefore, if one has moral objections to, say, abortion, this person should know which risks the profession entails in terms of moral injury. While this remains a valid and challenging point against a right to conscientious objection in healthcare, the focus on moral distress and moral injury might offer more scope for negotiation around it. After all, a healthcare professional might well be willing to sign up for things that conflict with their moral conscience (in the same way as one signs up to take additional health risks), but not to the type of moral injury that might follow. I might think I will be able psychologically and emotionally to cope with an abortion, or with helping someone to die medically, only to find out that I am emotionally and psychologically damaged by such practices. It is true that whether someone can cope psychologically with performing abortion is in many cases and to a certain extent independent of one’s moral stance on it. Many people who support abortion rights find the procedure psychologically and emotionally burdensome just because of what it involves, rather than because of its moral implications. However, the categories of moral distress and moral injuries capture precisely those cases where the ability to cope is related to one’s moral stance, and these are the ones this article wanted to bring attention to.

The social contract on which many views around healthcare professionals’ obligations are built is largely based on presumed consent to whatever interpretation is retroactively given to a vague notion of ‘duty of care’. But this might include unforeseen and unforeseeable situations that many would not accept if they were made explicit at the time of entering the profession. For one thing, it is impossible to foresee all the situations and risks a professional might face and to which the notion of ‘duty of care’ would be applied—and some might understand the consent given through an implicit social contract only to apply to procedures that are legal at the time one enters the profession, rather than to those that will become available and legal afterwards (an issue I am happy to remain neutral about in this context). For another thing, codes of practice are typically, and perhaps intentionally, vague, leaving room to various interpretations. The idea that a duty of care is a uniform, well-defined notion that establishes obligations for healthcare professionals in times of crises is challenged by the fact that ‘traditionally epidemics have not been met with the expectation that all doctors serve equally, but with the financing of cadres of ‘plague doctors’ or with the exploitation of existing pools of military medical personnel (in the influenza epidemic after the first world war) who are habituated to following orders and accepting risk’.^35^

It is unclear whether appeal to limits of a duty of care on the basis of risks of moral injury, moral distress and burnout would allow to provide more solid answers to the question whether healthcare professionals should have a right to conscientious objection. A proper argument would need to be formulated to the effect that moral injury or moral distress constitute psychosocial hazards related to the healthcare profession which can legitimately limit the scope of a duty of care. However, this seems to me the most challenging argument someone opposed to a right to conscientious objection in healthcare would need to face.

## Conclusion

Focusing on a principle of freedom of conscience or on the value of moral integrity to ground a right to conscientious objection in healthcare has resulted in a debate that has reached an impasse. There simply is not enough common ground between proponents and opponents of a right to conscientious objection to move the debate forward. At the core, there is an apparently unresolvable disagreement around two very complex issues: the nature of healthcare delivery (and what kind of individual professionals’ discretion it requires) and the relative moral weight of freedom of conscience and professionalism.

I have suggested that rethinking a claim to conscientious objection as a claim to avoid psychological harms in terms of moral distress or moral injury, rather than as an expression of freedom or as a way of upholding the value of moral integrity, is a more promising way of approaching the debate. That is because unlike appeals to freedom of conscience or to what counts as appropriate healthcare, the demandingness of certain tasks represents a common currency in debates around professional obligations. Everyone agrees professionals should not be expected to do what is too demanding for them, and the debate is about what counts as too demanding. If risks or burdens for one’s health legitimately constrain a duty of care, we should start thinking about whether moral injury and moral distress resulting from acting against one’s moral beliefs qualify for inclusion in that same category. This requires, among other things, finding a way of assessing such burdens and an adequate conception of demandingness that is applicable to acting against one’s conscience. Perhaps something to which more research should be dedicated.

## Data Availability

No data are available.
